# Barriers to teaching communication skills in Spanish medical schools: a qualitative study with academic leaders

**DOI:** 10.1186/s12909-020-1944-9

**Published:** 2020-02-10

**Authors:** Roger Ruiz Moral, Cristina García de Leonardo, Alvaro Cerro Pérez, Fernando Caballero Martínez, Diana Monge Martín

**Affiliations:** 1 Department of Medical Education, School of Medicine, Faculty of Health Sciences, Universidad Francisco de Vitoria (UFV), Edificio E. Ctra M-515 Pozuelo-Majadahonda, 3028 Madrid, Spain; 2 Neurophysiologist, Physiology, School of Health Sciences (UFV), Madrid, Spain; 3 School of Medicine, Faculty of Health Sciences (UFV), Madrid, Spain; 4 Family Physician, Epidemiology and Statistics, School of Health Sciences (UFV), Madrid, Spain; 5 Family and Preventive Medicine, Epidemiology and Statistics, School of Health Sciences (UFV), Madrid, Spain

**Keywords:** Communication skills, Medical students, Educational barriers, Medical education, Qualitative study, Teaching methods, Undergraduate studies, Medical school

## Abstract

**Background:**

In recent years, Spanish medical schools (MSs) have incorporated training in communication skills (CS), but how this training is being carried out has not yet been evaluated.

**Objective:**

To identify the barriers to the introduction and development of CS teaching in Spanish MSs.

**Methods:**

In a previous study, 34 MSs (83% of all MSs in Spain) were invited to participate in a study that explored the factual aspects of teaching CS in these schools. The person responsible for teaching CS at each school was contacted again for this study and asked to respond to a single open-ended question. Two researchers independently conducted a thematic analysis of the responses.

**Results:**

We received responses from 30 MSs (85.7% of those contacted and 73% of all MSs in Spain). Five main thematic areas were identified, each with different sub-areas: negative attitudes of teachers and academic leaders; organisation, structure and presence of CS training in the curriculum; negative attitudes of students; a lack of trained teachers; and problems linked to teaching methods and necessary educational logistics.

**Conclusions:**

The identified barriers and problems indicate that there are areas for improvement in teaching CS in most Spanish MSs. There seems to be a vicious circle based on the dynamic relationship and interdependence of all these problems that should be faced with different strategies and that requires a significant cultural shift as well as decisive institutional support at the local and national levels. The incorporation of CS training into MS curricula represents a major challenge that must be addressed for students to learn CS more effectively and avoid negative attitudes towards learning CS.

## Background

Research carried out over the last few decades has shown that good clinical communication (CC) has a positive influence on many clinical outcomes and aspects related to the patient and doctor, including physiological results, changes in health behaviour, the clinical relationship, healthcare procedures and the economic impact of healthcare [1–3]. There is also scientific evidence that clinical communication skills (CS) can be learned by students, doctors and other healthcare professionals [4, 5]. As a result, CS training has for some time been incorporated into undergraduate medical studies in a number of different countries where published guidelines describe the most appropriate and efficient strategies for developing CS within course curricula [6–9]. These guidelines are based on the available scientific evidence, and recommendations indicate that teaching 1) must be longitudinally sustained throughout the curriculum to improve content retention [10, 11]; 2) must include “experiential” teaching methods (role-playing, practice with simulated patients, observation, feedback, and small group discussions) [12, 13]; 3) must be integrated within the general medical curriculum and practical activities of medical specialties [14, 15]; and 4) in simulation scenarios and medical clerkships, must offer the opportunity to practice these skills and receive feedback to all students. To be effective, explicit learning and training objectives must be established that allow teachers to conduct directed observations and provide relevant feedback. In Spain, a white book from the National Agency for Quality Assessment and Accreditation (ANECA) referred to CS as a specific competence to be taught in MSs [16]. Furthermore, two years later, an order by the Ministry of Education and Science establishing the requirements for certifying an official university degree in medicine defined CS as one of seven competencies that medical students must acquire to obtain a medical degree [17]. Both documents provided a generic, declarative perspective on the subject, leaving accredited schools free to organise their CS curricula, teaching methods and assessments.

In 2017, a study was conducted by our group with the participation of 83% (34) of Spanish MSs to explore the current circumstances of CS teaching in Spanish MSs [18]. The study concluded that while almost all MSs had formally incorporated some content related to CC into their curricula, the evidence suggested that, in the vast majority of cases, CS teaching did not follow many of the main recommendations of the aforementioned bodies. As a result, while debate continues about many aspects of CC training, efforts to incorporate it into undergraduate studies may not be effective for Spanish medical students to acquire these CS. Thus, the study showed that a very significant percentage of these MSs (64.7%) only offered CS training during preclinical years and that this training was taught separately from other clinical skills and instead alongside the more theoretical subjects of humanities, ethics, history and, above all, psychology. It was also noted that there were no structured CS training programmes, that is, programmes with clear learning objectives and formative and summative assessment strategies, at any stage of the curriculum. In 29.5% of the MSs, this “training“ was considered part of the medical clerkship and had no clear learning objectives. The predominant teaching method was a traditional class format or seminars in smaller groups where the use of role play or simulated patients (SPs; a scarcely used resource) was carried out in a predominantly demonstrative manner. Thus, the majority of students did not have the opportunity to practise individually or to receive structured and constructive feedback on their performance. The study also revealed that although the majority of teachers were clinicians, they lacked specific training in this subject, while a significant number of teachers had no clinical experience or had no specialities outside of standard clinical practice, especially in psychology. Finally, CS were generally assessed using the traditional method of a written exam with very little structured assessment of student performance in simulated or actual settings.

As a result, to extend the study, we proposed a new exploration of the current realities of CS training in Spanish MSs, inviting the same academic leaders who participated in the previous study to offer their opinions on the problems and barriers to incorporating and implementing CS training in their own MSs. The aim of this second survey was to analyse the current situation of the CS training examined in the previous study to identify the main problems faced by schools as well as the priority areas for future action to make training more effective.

## Methods

This is a qualitative study that aims to explore the principal problems and barriers to the development of CC training in Spanish medical schools (MSs) based on the opinions of the professors responsible for teaching this domain in the curriculum.

In the preliminary study, 41 MSs, which were drawn from the official list of Spanish MSs according to the Ministry of Health in 2017, were invited to participate (http://todofp.es/que-como-y-donde-estudiar/que-estudiar/nuevos-titulos.html). A total of 34 MSs, or 83% of those contacted, replied (28 public and 6 private schools) and provided information on their curricula (credits, subject/s and year/s of teaching; methodology; etc.), characteristics of the faculty and the teaching and assessment methods used in CC training [18]. Two researchers (CGL and ACP) contacted the schools three times over a period of three months (from October to December 2018) and collected the data. Contact with the MSs was mainly carried out by email and by telephone for those who had not yet responded. Most of the MSs had a well-positioned contact person (who was responsible for CS training in the curriculum), while in other MSs, the dean provided information directly, without noting the position of the staff member directly in charge. Then, the 34 MSs that participated in the first stage of the survey were again contacted and invited to participate in the qualitative study (excluding the 7 MSs that did not initially respond). Participants were asked to respond to the following single, open question developed for this study: *“What have been (or are) the main barriers to including (in the past) and improving (in the future) student training in doctor-patient communication as part of the undergraduate degree offered by your school?“* There were not any prompts following this single question.

Analysis: All results were codified thematically and independently by two researchers, RR and AC. The results were sorted into thematic categories and subcategories, and there was a high degree of agreement between categories. There was a discrepancy in the allocation of subcategories, but this discrepancy was resolved through discussion to illustrate varying opinions, perspectives and agreements. The most relevant themes emerging from the results are presented below, including the number of times each theme appeared and the statements that best illustrate the opinions of the staff.

## Results

Of the 34 MSs that participated in the previous study and were contacted for this qualitative study, we received responses from 30 (85.7% of those contacted and 73% of all MSs in Spain; 5 private schools). Five main thematic categories were identified, each with different subcategories. Table 1 provides a list of the barriers identified:

**Table 1:** Barriers to teaching/learning communicative skills in Spanish medical schools

Negative attitudes of teachers and academic leaders (as a result of opinions such as...) (23 comments)	CS are not practically used The material used to teach CS is not scientific CS are innate skills CS cannot be taught The introduction of CS training threatens both teachers’ subjects and their own academic statuses
Marginal presence in the curriculum: organisation and structure (30 comments)	CS training is incorporated as a theoretical subject in an ad hoc style during preclinical periods CS training is incorporated in a fragmented way (in different subjects) CS training is included in subjects with other non-clinical content (humanities, ethics, history of medicine, psychology) There is no transversal structure with coherent teaching aims
Negative student attitudes (as a result of opinions such as...) (11 comments)	Students do not understand the use of CS CS training includes reductionist and scientific epistemological interpretations CS training is not important because it is not assessed CS training is not useful for the MIR (medical intern) exam CS training is not important because it is of a marginal or secondary nature in the curriculum CS are innate and subjective and cannot be learned
Limited and poorly trained teaching staff (13 comments)	There are no teachers with an appropriate academic status The clinicians use a weak or negative model Teachers have no training in CS or teaching methods
Teaching and assessment methods needed (21 comments)	Teachers do not use experience-based teaching methods Experience-based methods are expensive CS training requires more time CS training requires continuity and the commitment of teaching staff CS training requires a relatively sophisticated infrastructure CS training requires complex assessment systems that are not necessarily well known

### Negative attitudes of the university professors and academic leaders

The comments under this category illustrated that the principal barrier is the negative attitudes of academic leaders and university professors who teach traditional subjects and their influence on the way the CS curriculum is incorporated and structured.

Respondents’ perceptions of the responsibility for CS curricula indicated that academic leaders feel that CS are not very useful:

*“The inclusion of CC as an interdisciplinary subject or skill was outlandish and unnecessary, taking time away from real teaching”.* (U-5)

*“Teachers of unrelated subjects or content believe it to be ‘superfluous’, ‘not very serious’, ‘lacking content’, etc.”* (U-11)

Some respondents who were responsible for the curricula felt that some university professors think that there is no scientific (biomedical) evidence to support teaching CS:

*“Many teachers and board members… believe that the important thing in medicine is basic research and medical knowledge in order to get a good result on the MIR exam, so subjects like this distract students from what is important”.* (U-21)

*“It's believed to be a ‘soft science’ by the academic and professional community, who are more interested in technology”.* (U-22)

Other respondents who were responsible for the curricula thought that academic leaders do not perceive CS to not be useful but to be something that threatens their own teaching statuses:

*“There are a lot of people in the university, or ‘the establishment’, especially in the pre-clinics, who don't see the relevance of these skills in medicine. There are also clinicians rooted in a medical education model that dates back to the middle of the 19th century”.* (U-28)

*“Because of ignorance and a lack of understanding and consideration by academic leaders, most of whom are heads of departments and/or full-time lecturers, they don't value it and see it as an ‘easy subject’ that takes away teaching time from what is really important for them”.* (U-17)

Finally, some respondents who were responsible for the curricula believed that many academic leaders think that CS are learned by modelling:

*“The undergraduate degree administrators don't forcefully or confidently support training in these skills… as they consider it to be something that you learn through imitation”.* (U-10)

### Organisation, structure and presence of CS training in the curriculum

Comments under this category were the most frequent and repeated comments by key respondents; these comments suggest that CS training is primarily included as a legal obligation and is therefore implemented without an adequate teaching plan:

Incorporated in an ad hoc, theoretical way during preclinical periods:

*“It's (CC is) covered in an ad hoc way as part of another subject, preclinical psychology, where it's taught in a theoretical manner with no practical training alongside other clinical skills that are developed during clinical periods”.* (U-16)

Incorporated where it is easiest, i.e., together with other secondary subjects:

*“... it (CC) is crammed in, given no time of its own, with legal medicine, bioethics, …and at different points in time, which makes it difficult to organise. It seems as though there is no other way to incorporate these skills, and so they are crammed in where there are a few credits leftover”.* (U-17)

Incorporated in a fragmented way with no coherent framework that includes objectives:

*“...as it's not a ‘respected’ skill by academic leaders, it's only covered in an ad hoc and very limited way as part of smaller subjects, often optional, within different clinical and practical subjects, but with no specific objectives (as though the student would be able acquire them ‘by magic’)...it's a genuinely ‘orphaned skill’”.* (U-26)

Need to be incorporated into curricula in a structured, transversal way at a supra-departmental level:

*“An institutional or structural barrier is that curricula do not incorporate the subject in an obvious way. In general, they recognise the need for it but do not explain how it will be carried out and where the necessary credits will come from”.* (U-23)

### Negative student attitudes

The respondents identified student attitudes towards CC as a major barrier. They linked these attitudes to a number of different causes.

Negative attitudes towards CS because of a lack of understanding of why they are useful:

*“It's basically covered in second-year psychology when the majority of students are, in my opinion, not mature enough to understand the importance of this topic in their future clinical roles”.* (U-13)

*“The main barrier is that the subject is covered in second-year medicine, at the same time as the Golgi apparatus, cranial nerves and the Starling Law, so for the students, its use is relative, given that it will be at least another two years before they work with patients and can see why it's important to their work as doctors”.* (U-28)

Negative attitudes towards CS due to biomedical epistemological interpretations:

*“Many students believe that the education consists of gaining a lot of medical knowledge”.* (U-17)

*“Students tend to want to ‘objectivise’ all the assessment schemes (when tackling exam revision, trying to boost results and competing for grades). This makes an overall assessment of communicative skills difficult and entails going through meticulous and debatable evaluations”.* (U-11)

Negative attitudes towards CS training due to it not being assessed:

*“Although student attitudes have changed in the last few years... due to (CC) not being a continuous feature of a stable assessment scheme... they don't have enough motivation to study it”.* (U-23)

Negative attitudes towards CS training due to it not being useful for the MIR exam:

*“Medical students continue to have a pre-academic profile for the MIR exam that prioritises the absorption of knowledge... so it (CC) has a passive role in clerkships, with no or little feedback or reflection on their communication...they are demotivated”* (U-24)

Negative attitudes towards CS training because of how it is included and taught in curricula:

*“By including it (CS training) as something secondary within other subjects, generally pre-clinics, using inadequate teaching method, if any, and with no thought as to how it is assessed, students see it as something that is not very important or related to their own personality”* (U-26)

### Lack or absence of trained teachers

The comments in this category referenced the absence of trained teachers in regard to both adequately teaching the content (offering feedback, etc.) and adequately planning it in the curriculum:

*“There aren't enough trained associate teachers involved in this subject area to be able to establish proper parameters for communication skills, teaching objectives and teaching methods”.* (U-4)

*“There aren't enough trained teachers to teach it (CC) properly. It is left ‘in the hands’ of the teaching clinicians in charge of clerkships. The psychologists don't generally have trained teachers that know the clinic”.* (U-5)

### Problems linked to teaching methods and the necessary educational logistics for CS training to be taught

The comments under this category indicated that the teaching method designed specifically for teaching CC poses a significant barrier to CC assessment.

Technical/infrastructure requirements:

*“The type of teaching necessary: active learning environments, with simulated patients, video recordings, self-evaluation...”* (U-9)

*“It requires a specific infrastructure for it to be carried out, spaces for simulation, video recording and reproduction systems...”* (U-14)

Insufficient time:

*“The main barrier we face in communication workshops is, without a doubt, a lack of time...for students to individually put into practice what they have learned, give subsequent feedback on how to improve any error made in the practice interview with a simulated patient”.* (U-19)

Structured feedback:

*“Every student would have to be given personalised feedback while interacting with simulated or real patients”.* (U-23)

Continuity and commitment of teaching staff:

*“It's not thought that this type of learning needs to be continually incorporated throughout the degree. It's thought that by merely studying subjects such as psychology, oncology, palliative care or psychiatry, students will learn communication skills...in reality, during clerkships, which is when students are faced with communication problems, they really are alone. In general, there is no feedback given by teaching clinicians”.* (U-26)

High cost:

*“This subject would have to receive more investment than others: SP, Gesell chamber...”* (U-30)

Problems derived from the type of assessment that CS require:

*“Assessment makes it (CC) a major burden. Exams here are worthless; they (students) should be assessed on what they do, how they really communicate and not what they know”* (U-11)

*“The students have to take an objective test (a simulated exam with a standardised patient) for the skills they've acquired to be assessed...and this is difficult to carry out and expensive”.* (U-19)

## Discussion

In this qualitative study, individuals responsible for CS training in their MSs offered their personal opinions on the most significant problems or barriers to incorporating and developing these skills. These key informants agreed on the principal problems: the negative attitudes of teachers and academic leaders, who regard CS as marginal; little investment in educational methodologies and evaluative structures; limited training and promotion on the part of faculty; and the negative attitudes of students towards CS. These barriers have resulted in a teaching approach based on traditional teaching methodologies (lectures and workshops) and assessment strategies (written exams), poor curricular integration, and a theoretical approach that is often detached from clinical practice and taught by non-clinical professors. Although surveys have shown that educational input generally improves CS among trainees [13], teaching methods classified as “experiential” [19], including role play, simulated patients, supervised practical training, self-assessment and peer feedback, are more effective [4, 12, 20, 21]. Experiential learning is a structured cyclical learning process that occurs through awareness, practice, reflection and feedback (individually or as a group) delivered in a structured way by experienced professionals [19, 22, 23]. A focus on learning tasks and skills improves student acquisition of useful communication strategies for use with patients [4, 12, 20, 21]. In recent decades, these educational strategies have been incorporated at a varying scope and depth in the curricula of many MSs around the world. However, most schools have encountered barriers and problems similar to those identified by our key informants [24–28].

**Fig. 1:**
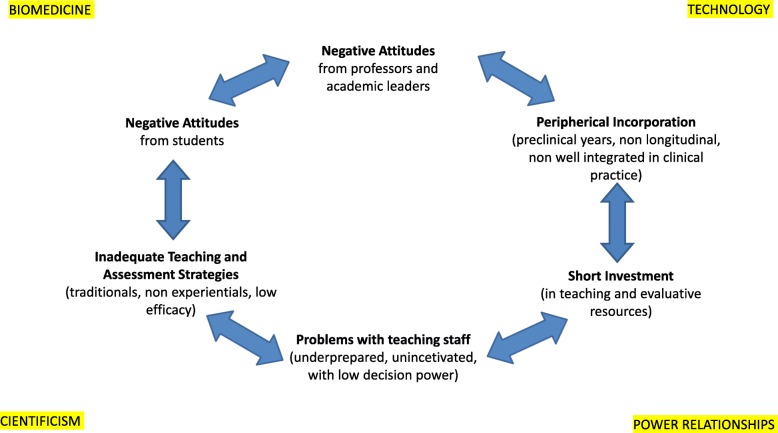
The Dynamics of Communication Skills (CS) Training in Spanish Medical Schools

The general opinions offered and the similarity of the statements by the key informants offer a perspective on the educational context in which the biomedical paradigm is hegemonic, revealing the effect that these barriers and dynamics have on the educational environment. In Fig. 1, we present these barriers in context to provide a better understanding of their dynamics and an opportunity to explore possible strategies to improve and progress towards a more efficient approach to teaching CS.

To varying degrees, many of the barriers have been identified in recent years. The beliefs that communication is innate, lacking in academic credibility, subjective and unscientific have been identified elsewhere [29, 30], not only among academic leaders and teachers of other subjects but also among many students [31]. This interpretation reveals the influence of the biomedical paradigm of academic medicine in Spain that determines what is appropriate and inappropriate to research, teach and, of course, practice. Several studies have shown the extent to which the introduction of humanities and social sciences in medical curricula is hindered by such hegemonic thinking [32–35]. This study highlights that, according to key respondents, this ideological hegemony is a determining factor in the incorporation of CS in MS curricula in Spain. Figure 1 shows the dynamics that link the different barriers as a vicious circle within the framework of this ideological influence. In effect, when taken as a whole, these barriers form a coherent and revelatory picture of the problem: in an educational environment dominated by biomedicine, the negative attitudes of teachers and academic leaders towards this subject are primarily due, according to the respondents, to their beliefs that CS are “not scientific“ and are innate skills. Other factors are also at work, including the position of universities within the political power structure of Spain. Thus, the legal requirement for the introduction of CS into MS curricula, as per the ministerial order of 2008, would appear to be temporary, peripheral or otherwise secondary, with CS training included within other preclinical subjects and without an integrated and coherent teaching plan. Furthermore, when CS are taught as a preclinical subject or in a theoretical way by teachers who are not clinicians, the tacit message to students is that clinicians know the fundamentals of medical practice, while others specialise in less scientific, psychosocial topics [36]. This approach to teaching CS also implies that CS are an objective in themselves rather than a tool for better patient care [37]. All of this dismisses the fact that CS enhance clinical encounters, facilitating the sharing of clinical information, negotiation and decision making between the doctor and patient.

Additionally, the absence of sophisticated (experiential) teaching methods and appropriate assessment strategies also represents a barrier, as learning appears to have little impact on students. This barrier is evident both in the lack of training and in the difficulty of and disinterest in investing the required resources. The absence of practical training based on observation and feedback is not, however, exclusive to the Spanish education system [38–40], nor is having an “informal“ CS training plan “without structure, sufficient time or defined objectives“ [41]. Even well-structured programmes can generate negative student perceptions if adequate teaching methods are not used [42]. It has been observed that students who receive less experiential CS training are less likely to consider communication to be a skill to be learned and used to improve clinical results [43]. The use of experiential training methods is recommended (role play, simulations, feedback, etc.) for both teachers and students [44]. However, students may view these experiential sessions negatively if they are not conducted in a safe, trusting and prejudice-free environment [31, 44].

The study shows that teacher training is key and that a lack of qualified or experienced teachers is a major barrier. In fact, clinicians interested in CS complain that they receive little training and are reluctant to teach and assess skills they themselves have not fully mastered [28]. Furthermore, this lack of teacher training also limits the skills that can be taught [45]. Teaching CS in clinical practice requires teachers who believe in CS and can demonstrate them [46]. This is particularly important since CS training will remain deficient if supervisors do not believe these skills to be essential for a clinician and/or believe that they cannot be taught [46]. Additionally, clinical supervisors tend to teach CS using role modelling in a very irregular and rarely explicit manner [47]. When clinical supervisors address communication problems with residents and students, they generally intervene as correctors, clinicians or trainers rather than as teachers [28]. Many clinical supervisors hope that by simply watching and listening, young doctors will recognise, accept and imitate desirable behaviour [48]. The risks of relying on role modelling for CS training during internships have been observed by clinical supervisors who have noted that models often lack the required skills [49]. Respondents also noted the absence of teachers in influential posts to champion CS training and push for changes to the curriculum.

The weight accorded to CS in the Spanish system and the way in which CC is assessed have proven to be counterproductive. In general, Spanish MSs do not always assess CS in a specific way, or they asses CS by using a written exam with multiple choice questions, a list of skills to be completed by the supervisor at the end of the internship or unsuitable checklists. These assessment methods give students the impression that these skills are unimportant or secondary and are not usually well received [50, 51].

Finally, students and young doctors are described as having negative attitudes towards CS and failing to learn CS. While some believe that communication is innate and subjective and cannot be taught [29, 30], personal factors also play a role. Students with the most positive attitude towards CS are generally women whose parents are not doctors or women who believe that their CS needs improvement [52]. Student attitudes also vary according to their level of experience. Younger doctors with less experience tend to be more stressed and less open to improving communication problems than those with more experience because they are still concerned about providing accurate diagnoses and providing quick and effective care [28]. Poor knowledge of biomedicine or clinical reasoning or a lack of skill in using technical procedures (electronic medical registers) [49, 53] can also hinder communication. However, while these factors doubtlessly contribute to a failure to see the benefits of CS in medicine, they may also confirm negative attitudes towards these skills while also reaffirming reductionist conceptions of medicine and CC, creating a vicious circle that is difficult to break (Fig. 1).

However, despite all of these barriers, particularly the negative attitudes of professors and academic leaders towards CS training, studies have shown that young doctors and students value CS when they are taught experientially using a student-centred approach and when clinical supervisors take a more active role in observing them and offering feedback [54, 55]. Young doctors and students have also come to expect training in clinical CS in MSs and residence programmes [56–58]. Student representatives in Spain have made these requests explicit [59].

## Conclusions

Individuals responsible for introducing and developing CS in Spanish MSs identified a set of barriers that make it difficult for students to effectively learn these skills; these barriers represent a set of interrelated problems that largely explain the way in which CS are being introduced and the real priority for them being taught in many of the Spanish MSs. Effectively incorporating CS teaching into MS curricula is a challenge that will require a significant cultural shift and support from all local and national academic and institutional levels as a prerequisite for introducing new educational models that are more in line with new teaching and clinical practice trends.

## Data Availability

All transcriptions of the survey responses and their qualitative analysis for the purposes of this study are available from the corresponding author upon request.

## Abbreviations

ACP: Alvaro Cerro Pérez
CC: Clinical Communication
CS: Communication Skills
MS: Medical School
RRM: Roger Ruiz Moral
SP: Simulated Patient
U: University
